# Supplementary material for: Development of chemokine network inhibitors using combinatorial saturation mutagenesis
Source: Commun Biol. 2025 Apr 3;8:549. doi: 10.1038/s42003-025-07778-6 (PMC11969024; doi:10.1038/s42003-025-07778-6)
Supplement: Supplementary file 4 — Supplementary Data 2 [file 42003_2025_7778_MOESM4_ESM.zip › SupplementaryData3/scripts/xattune_dr.tar!/./script/analysis/JK/JKSuppTableX.docx]

Supplementary Table X - Mean and standard error (SE) of chemokine pEC50 and
pEC80 values

| Chemokine | Cells | Replicates | Mean pEC80 [M] | SE pEC80 [M] | Mean pEC50 [M] | SE pEC50 [M] |
| --- | --- | --- | --- | --- | --- | --- |
| CCL2 | THP1 | 3 | 8.35 | 0.51 | 8.73 | 0.51 |
| CCL3 | THP1 | 2 | 9.31 | 0.02 | 9.64 | 0.02 |
| CCL5 | THP1 | 7 | 8.36 | 0.03 | 8.65 | 0.03 |
| CCL7 | THP1 | 4 | 8.02 | 0.12 | 8.37 | 0.12 |
| CCL8 | THP1 | 7 | 7.84 | 0.04 | 8.19 | 0.04 |
| CCL14 | THP1 | 4 | 9.23 | 0.04 | 9.61 | 0.04 |
| CCL19 | ATC | 6 | 7.64 | 0.27 | 7.91 | 0.27 |
| CCL21 | ATC | 3 | 7.42 | 0.20 | 7.98 | 0.20 |
| CCL23 | THP1 | 3 | 8.66 | 0.11 | 8.97 | 0.11 |
| CXCL6 | J:CXCR1 | 7 | 7.45 | 0.08 | 7.71 | 0.08 |
| CXCL9 | ATC | 5 | 7.22 | 0.19 | 7.40 | 0.19 |
| CXCL10 | ATC | 3 | 7.50 | 0.05 | 7.87 | 0.05 |
| CXCL11 | ATC | 3 | 7.99 | 0.52 | 8.25 | 0.52 |
| CXCL12 | ATC | 3 | 8.54 | 0.05 | 8.93 | 0.05 |
| ATHERO | THP1 | 4 | 8.29 | 0.04 | 8.73 | 0.04 |
| ATHERO | ATC | 5 | 7.39 | 0.18 | 7.84 | 0.18 |
| INSULITIS | THP1 | 4 | 7.22 | 0.08 | 7.75 | 0.08 |
| INSULITIS | ATC | 3 | 7.17 | 0.04 | 7.53 | 0.04 |
| RA | THP1 | 4 | 7.72 | 0.06 | 8.20 | 0.06 |
| RA | ATC | 3 | 7.42 | 0.06 | 7.87 | 0.06 |
